# Effect of the Interaction Between Outdoor Air Pollution and Extreme Temperature on Daily Mortality in Shanghai, China

**DOI:** 10.2188/jea.JE20110049

**Published:** 2012-01-05

**Authors:** Yuexin Cheng, Haidong Kan

**Affiliations:** 1 Department of Hematology, Fourth Affiliated Hospital of Nantong University, First Hospital of Yancheng, Yancheng, China; 2 School of Public Health, Key Lab of Public Health Safety of the Ministry of Education, Fudan University, Shanghai, China; 3 G_RI^o^CE (Research Institute for the Changing Global Environment) and Fudan Tyndall Centre, Fudan University, Shanghai, China

**Keywords:** air pollution, climate change, extreme temperature, interaction, time-series

## Abstract

**Background:**

Both outdoor air pollution and extreme temperature have been associated with daily mortality; however, the effect of their interaction is not known.

**Methods:**

This time-series analysis examined the effect of the interaction between outdoor air pollutants and extreme temperature on daily mortality in Shanghai, China. A generalized additive model (GAM) with penalized splines was used to analyze mortality, air pollution, temperature, and covariate data. The effects of air pollutants were stratified by temperature stratum to examine the interaction effect of air pollutants and extreme temperature.

**Results:**

We found a statistically significant interaction between PM_10_/O_3_ and extreme low temperatures for both total nonaccidental and cause-specific mortality. On days with “normal” temperatures (15th–85th percentile), a 10-µg/m^3^ increment in PM_10_ corresponded to a 0.17% (95% CI: 0.03%, 0.32%) increase in total mortality, a 0.23% (0.02%, 0.44%) increase in cardiovascular mortality, and a 0.26% (−0.07%, 0.60%) increase in respiratory mortality. On low-temperature days (<15th percentile), the estimates changed to 0.40% (0.21%, 0.58%) for total mortality, 0.49% (0.13%, 0.86%) for cardiovascular mortality, and 0.24% (−0.33%, 0.82%) for respiratory mortality. The interaction pattern of O_3_ with lower temperature was similar. The interaction between PM_10_/O_3_ and lower temperature remained robust when alternative cut-points were used for temperature strata.

**Conclusions:**

The acute health effects of air pollution might vary by temperature level.

## INTRODUCTION

Short-term exposure to outdoor air pollution has been linked to adverse health effects, including increased mortality, higher rates of hospital admissions and emergency department visits, exacerbation of chronic respiratory conditions (eg, asthma), and decreased lung function.^1^ Temperature can also affect human health. The association between daily mortality and temperature has been extensively observed.^2^ Typically, a U-shaped relationship between mortality risk and temperature level is noted: mortality risk decreases from the lowest temperature to an inflection point and then increases with higher temperature.^3^ Basu and Samet maintained that the effect of temperature on morality might differ in areas with different weather patterns, latitudes, air pollution levels, and prevalence of air-conditioning systems.^2^

The rapid buildup of greenhouse gases is expected to increase not only mean temperature but also temperature variability. This change adds urgency to the need to better understand the health impact of extreme temperature, as well as its interaction with other environmental stresses.^4^^,^^5^ The interaction between outdoor pollution and extreme temperature was investigated as early as 1972.^6^ Since then, however, only a few studies have examined this issue. Katsouyanni et al analyzed the potential interaction between air pollution and high temperature and found evidence of an interaction effect.^7^ Roberts suggested that the interaction between daily particulate air pollution and daily mean temperature should be considered in epidemiologic studies of air pollution.^8^ Recent analyses of the season-specific effects of air pollution highlight the need for comprehensive investigation of the interaction between air pollution and temperature, because season is obviously related to temperature.^9^^–^^14^

Epidemiologic evidence of an interaction between air pollution and extreme temperature is scarce and the question remains unanswered. In this time-series analysis we examined the effect of the interaction between outdoor air pollutants—ie, particulate matter less than 10 µm in diameter (PM_10_), sulfur dioxide (SO_2_), nitrogen dioxides (NO_2_), and ozone (O_3_)—and extreme temperature on daily mortality in Shanghai, China.

## METHODS

### Data

Shanghai, the most populous city in China, is located at the tip of the Yangtze River Delta in eastern China. The city has a moderate subtropical climate, with 4 distinct seasons and abundant rainfall. The demographic characteristics of our study population have been described elsewhere.^11^^,^^14^

In this analysis, daily mortality data (excluding injuries and accidents) for residents living in the 9 urban districts of Shanghai from 1 January 2001 through 31 December 2004 were collected from the database of the Shanghai Municipal Center of Disease Control and Prevention (SMCDCP), which is the government agency in charge of health data collection in Shanghai. Consistent with previous literature,^15^ we did not analyze deaths from injuries or accidents because they are assumed to be unrelated to air pollution. The death reporting system in Shanghai was implemented in 1951 and has been computerized since 1990. In Shanghai, all deaths must be reported to appropriate authorities before cremation. Physicians complete the death certificate cards for both in-home and in-hospital deaths. The information on the cards is then sent to SMCDCP through their internal computer network. As required by law, the causes of death for 2001 and 2002–2004 were coded according to the International Classification of Diseases, Revision 9 (ICD-9) and Revision 10 (ICD 10), respectively. In addition to total nonaccidental deaths (ICD-9, <800; ICD-10, A00–R99), mortality data were classified into deaths due to cardiovascular diseases (ICD-9, 390–459; ICD-10, I00–I99) and respiratory diseases (ICD-9, 460–519; ICD-10, J00–J98).

Daily data on levels of PM_10_, SO_2_, NO_2_, and O_3_ from 1 January 2001 through 31 December 2004 in metropolitan Shanghai were retrieved from the database of the Shanghai Environmental Monitoring Center (SEMC). The daily concentrations of each pollutant were averaged from the available monitoring results of 6 fixed-site stations located in urban areas of Shanghai and overseen by China National Quality Control. We abstracted the 24-hour average concentrations of PM_10_, SO_2_, NO_2_, and 8-hour (from 10 AM to 6 PM) average concentration of O_3_. The maximum 8-hr mean was used because the World Health Organization (WHO) has indicated that the 8-hr mean reflects the greatest health-relevant exposure to O_3_.^16^ When calculating the 24-hour average concentrations of PM_10_, SO_2_, and NO_2_ and the 8-hour average O_3_ concentration, at least 75% of the 1-hour values had to be available for that particular day.

We obtained daily meteorological data (including minimum, maximum, and mean temperature and relative humidity) from the Shanghai Meteorological Bureau. The weather data were measured at a fixed-site station located in the Xuhui District of Shanghai.

Data on mortality, pollutants, and meteorological variables were validated by an independent auditing team, which checked a sample of the original death certificates and monitoring records and validated the process for generating data on mortality, weather, and air pollution used in this time-series analysis.

### Statistical analysis

We used a generalized additive model (GAM) with penalized splines to analyze data on mortality, air pollution, and covariates. Because daily mortality counts typically follow a Poisson distribution, the core analysis was a GAM with log link and Poisson error, which accounted for smooth fluctuations in daily mortality. Consistent with several recent time-series studies,^17^^–^^19^ we used the penalized spline model in our analysis.

We first built the basic models for various mortality outcomes without including air pollution or weather variables. We incorporated smoothed spline functions of time, which accommodate nonlinear and nonmonotonic patterns between mortality and time, thus offering a flexible modeling tool. The partial autocorrelation function (PACF) was used to guide the selection of *df* until the absolute values of the sum of PACF for lags up to 30 reached a minimum. In this way, 4, 4, and 5 *df* per year were used for the time trend in our basic models for total, cardiovascular, and respiratory mortality, respectively. Day of the week (DOW) was included as a dummy variable in the basic models. Residuals of the basic models were also examined to check whether there were discernable patterns and autocorrelation by means of residual plots and partial autocorrelation function (PACF) plots.

After we established the basic models, we introduced the pollutant and weather variables and analyzed their effects on mortality outcomes. Based on the previous literature,^20^^–^^22^ 3 *df* (whole period of study) for mean temperature and relative humidity could satisfactorily control for their effects on mortality and was thus used in the model.

To examine the effect of the interaction between air pollutants and extreme temperature, we stratified the effects of air pollutants by temperature. As compared with other methods used to detect interaction effects, temperature stratification requires fewer parameters and yields a simple, quantitative comparison of the estimated effects of pollutants in various temperature strata.^8^ As in a prior study,^8^ we set the upper (U) and lower (L) temperature cut-points equal to the 85th and 15th percentiles of temperature, respectively. Due to this inherently arbitrary choice of cut-point values, a sensitivity analysis was performed to address the sensitivity of the estimated effects of air pollutants to the choice of cut-point values (95th and 5th, 90th and 10th, 80th and 20th, 75th and 25th). We tested the statistical significance of differences between effect estimates of the temperature strata (eg, the effect of PM_10_ on “normal” temperature vs low temperature days) by calculating the 95% confidence interval (95% CI) as $({\overset{⌢}{Q}}_{1}-{\overset{⌢}{Q}}_{2})\pm 1.96\sqrt{{\left(S{\overset{⌢}{E}}_{1}\right)}^{\text{2}}+{\left(S{\overset{⌢}{E}}_{2}\right)}^{\text{2}}}$, where ${\overset{⌢}{Q}}_{1}$ and ${\overset{⌢}{Q}}_{2}$ are the estimates for the 2 categories and $S{\overset{⌢}{E}}_{1}$ and $S{\overset{⌢}{E}}_{2}$ are their respective standard errors.^23^

To graphically illustrate the interaction between air pollution and extreme temperature, we fitted nonparametric response surface models to identify the joint effects of air pollution and temperature on daily mortality. We used a GAM to fit a response surface that captured the relation between the 2 main independent variables and the dependent variable, without assuming linearity.^24^^,^^25^ All analyses were conducted using R 2.10.1 and the MGCV package.

## RESULTS

**Figure 1.**
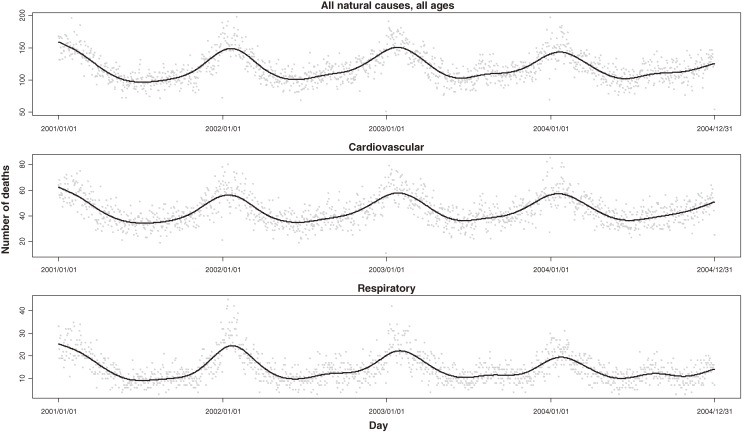
Time-series of total nonaccidental, cardiovascular, and respiratory mortality in Shanghai, 2001–2004. Solid lines are smoothing splines with 5 df/yr.

Our research population included approximately 6.3 million residents, and the number remained stable during our research period. From 2001 through 2004 (1461 days), 173 911 nonaccidental deaths were recorded in the study population. On average, there were 119.0 nonaccidental deaths per day in the target population, including 44.2 from cardiovascular diseases and 14.3 from respiratory diseases (Figure 1 ). Cardiopulmonary diseases accounted for 49.1% of all nonaccidental deaths among urban residents of Shanghai.

**Figure 2.**
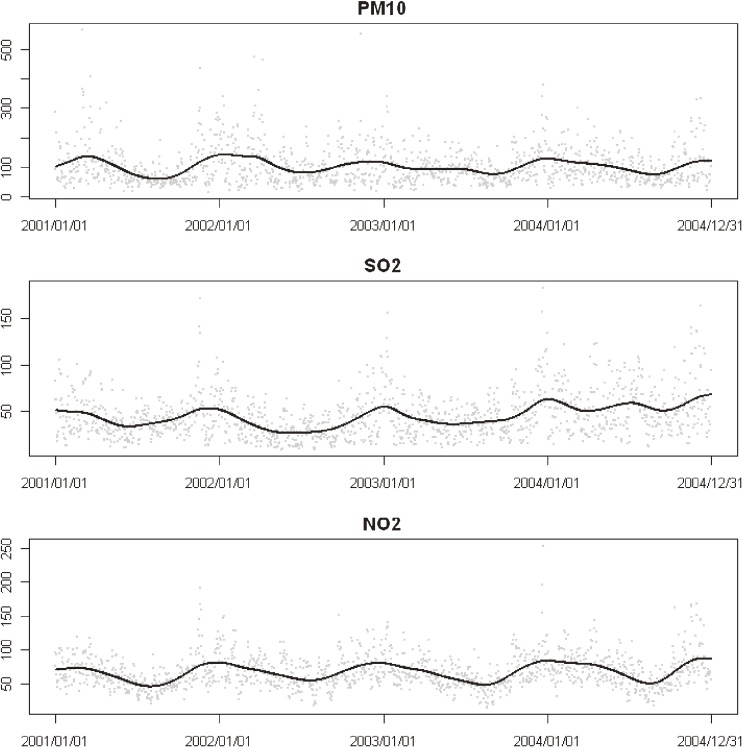
Time-series of monitor-averaged pollutant concentrations (µg/m^3^) in Shanghai, 2001–2004. Solid lines are smoothing splines with 5 df/yr.

**Figure 3.**
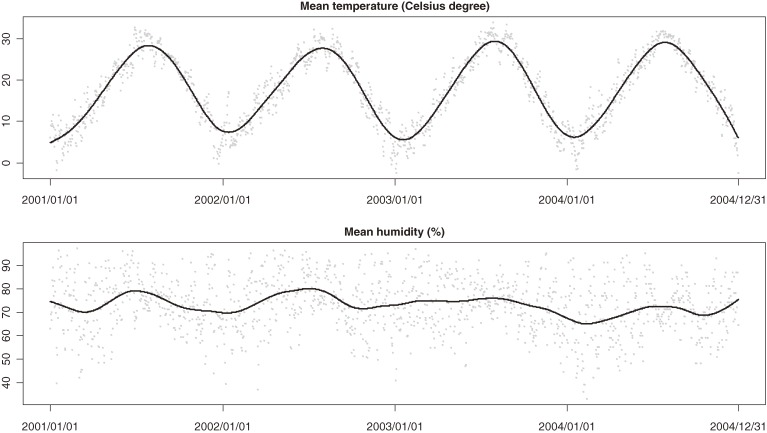
Time-series of temperature (°C) and relative humidity (%) in Shanghai, 2001–2004. Solid lines are smoothing splines with 5 df/yr.

The mean air pollution levels were 101.9 µg/m^3^ for PM_10_, 44.7 µg/m^3^ for SO_2_, 66.6 µg/m^3^ for NO_2_, and 63.5 µg/m^3^ for O_3_ (Figure 2 ). The data were 100% complete for all variables except O_3_ (7 missing days). Meanwhile, the mean daily average temperature and humidity were 17.7°C and 72.9%, reflecting the subtropical climate in Shanghai (Figure 3 ).

**Figure 4.**
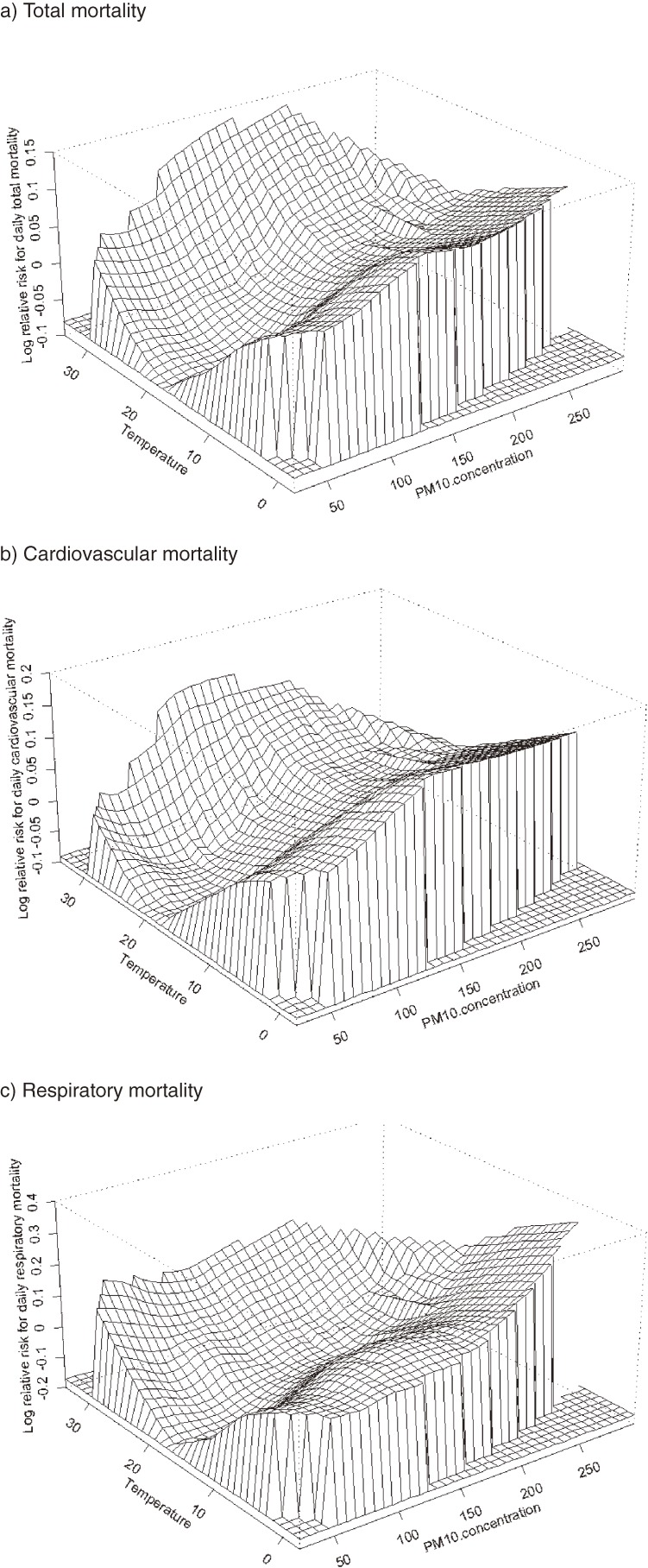
Bivariate response surfaces of temperature and PM_10_ for total (a), cardiovascular (b), and respiratory mortality (c) in Shanghai, 2001–2004.

**Figure 5.**
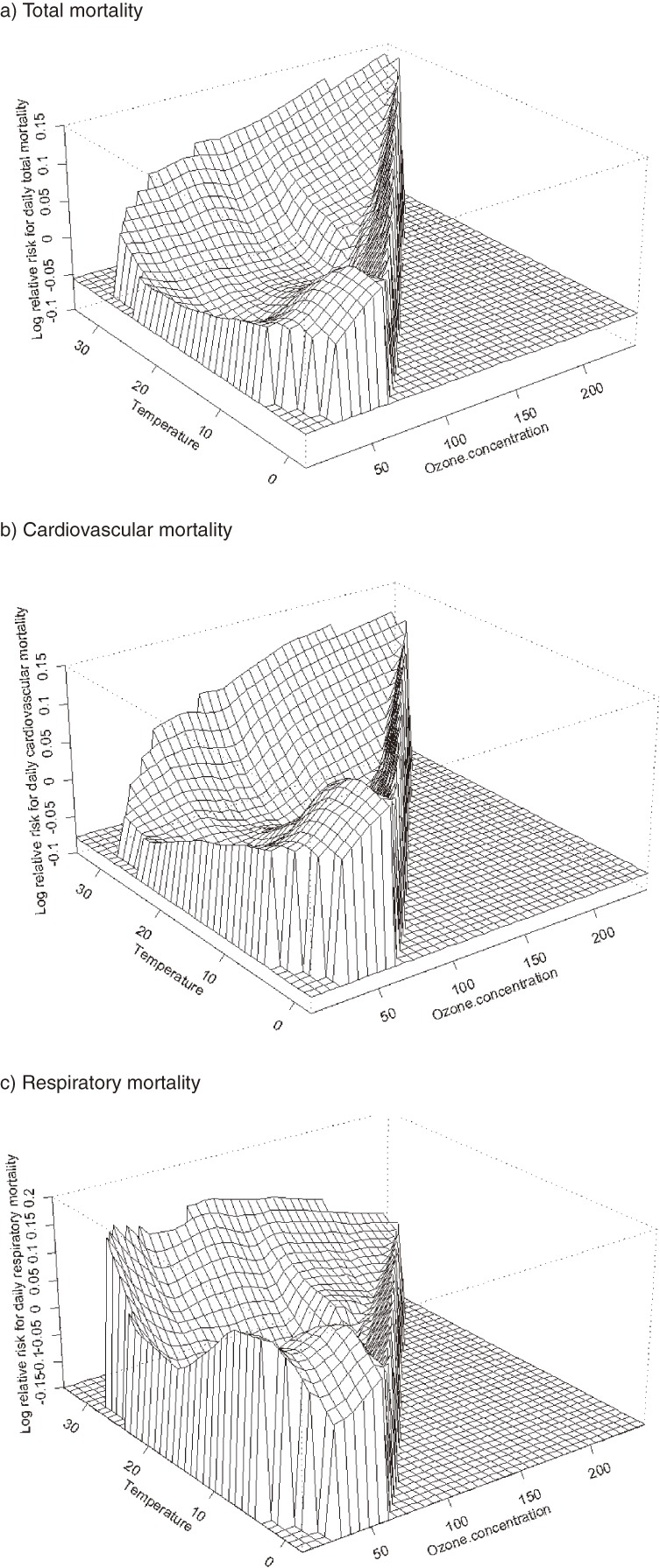
Bivariate response surfaces of temperature and O_3_ for total (a), cardiovascular (b), and respiratory mortality (c) in Shanghai, 2001–2004.

Figures 4 and 5 show joint response surfaces that illustrate the potential interactive effects of PM_10_/O_3_ and temperature on total, cardiovascular, and respiratory mortality. Tables 1 to 3 describe the results of a regression analysis of air pollutants stratified by temperature stratum. In general, the effect of the interaction between PM_10_ and extreme low temperature was statistically significant for both total and cause-specific mortality. For example, on “normal” temperature (15th–85th percentile) days, a 10-µg/m^3^ increment in PM_10_ corresponded to a 0.17% (95% CI: 0.03%, 0.32%) increase in total mortality, a 0.23% (0.02%, 0.44%) increase in cardiovascular mortality, and a 0.26% (−0.07%, 0.60%) increase in respiratory mortality. On low-temperature (<15th percentile) days, the estimates increased to 0.40% (0.21%, 0.58%) for total mortality (*P* = 0.01 compared with normal temperature days), 0.49% (0.13%–0.86%) for cardiovascular mortality (*P* = 0.04), and 0.24% (−0.33%, 0.82%) for respiratory mortality (*P* = 0.04). On high-temperature (>85th percentile) days, the numbers were 0.30% (−0.01%, 0.63%), 0.30% (−0.17%, 0.79%), and 0.35% (−0.49%, 1.19%) for total, cardiovascular, and respiratory mortality respectively, and none of these estimates significantly differed from those for normal temperature days. The interaction pattern of O_3_ with low temperature was similar (Tables 1–3). There was no significant interaction for SO_2_ or NO_2_. The use of alternative cut-points for temperature strata yielded similar trends.

**Table 1.** Percent change (95% CI) in total mortality per 10-µg/m^3^ increment in air pollutants at different temperature strata

Percentile cut-points for temperature (L, U)		PM_10_		SO_2_		NO_2_		O_3_	
			
		%	*P* value^a^	%	*P* value^a^	%	*P* value^a^	%	*P* value^a^
(5–95)	5% L	0.46 (0.21, 0.72)	0.03^b^	1.56 (0.95, 2.16)	0.05	1.25 (0.71, 1.79)	0.15	1.78 (0.84, 2.73)	<0.01^b^
	95% U	0.44 (0.07, 0.82)	0.18	1.05 (0.31, 1.79)	0.94	1.04 (0.33, 1.75)	0.73	0.54 (0.15, 0.92)	0.77
	5%–95%	0.20 (0.06, 0.34)		1.03 (0.63, 1.43)		0.92 (0.54, 1.31)		0.58 (0.25, 0.92)	
(10–90)	10% L	0.42 (0.20, 0.64)	0.04^b^	1.30 (0.78, 1.82)	0.29	1.17 (0.69, 1.66)	0.20	2.06 (1.21, 2.91)	<0.01^b^
	90% U	0.39 (0.02, 0.76)	0.29	0.91 (0.15, 1.67)	0.70	0.95 (0.23, 1.66)	0.95	0.47 (0.08, 0.86)	0.39
	10%–90%	0.19 (0.05, 0.33)		1.04 (0.63, 1.46)		0.93 (0.54, 1.32)		0.62 (0.29, 0.96)	
(15–85)	15% L	0.40 (0.21, 0.58)	0.01^b^	1.31 (0.84, 1.77)	0.16	1.20 (0.77, 1.64)	0.05	2.17 (1.46, 2.88)	<0.01^b^
	85% U	0.30 (−0.01, 0.63)	0.41	0.78 (0.05, 1.52)	0.52	0.81 (0.20, 1.42)	0.78	0.42 (0.05, 0.79)	0.19
	15%–85%	0.17 (0.03, 0.32)		1.01 (0.58, 1.44)		0.89 (0.49, 1.29)		0.66 (0.32, 1.00)	
(20–80)	20% L	0.30 (0.13, 0.47)	0.12	1.18 (0.73, 1.63)	0.54	1.07 (0.65, 1.50)	0.25	1.66 (1.03, 2.29)	<0.01^b^
	80% U	0.36 (0.07, 0.65)	0.20	1.03 (0.34, 1.71)	0.93	0.99 (0.44, 1.55)	0.70	0.51 (0.16, 0.85)	0.55
	20%–80%	0.18 (0.03, 0.33)		1.05 (0.62, 1.49)		0.90 (0.50, 1.30)		0.61 (0.25, 0.96)	
(25–75)	25% L	0.32 (0.15, 0.49)	0.05	1.20 (0.76, 1.64)	0.43	1.09 (0.66, 1.51)	0.22	1.44 (0.88, 2.00)	<0.01^b^
	75% U	0.29 (0.02, 0.56)	0.35	0.91 (0.25, 1.57)	0.68	0.93 (0.41, 1.45)	0.92	0.47 (0.13, 0.80)	0.46
	25%–75%	0.16 (0.01, 0.32)		1.04 (0.59, 1.49)		0.91 (0.50, 1.31)		0.59 (0.22, 0.96)	

Abbreviations: PM_10_, particulate matter less than 10 microns in diameter; SO_2_, sulfur dioxide; NO_2_, nitrogen dioxides; O_3_, ozone.

^a^*P* value for lower and upper temperature stratum compared with normal temperature.

^b^Statistically significant (*P* < 0.05).

**Table 2.** Percent change (95% CI) in cardiovascular mortality per 10-µg/m^3^ increment in air pollutants at different temperature strata

Percentile cut-points for temperature (L, U)		PM_10_		SO_2_		NO_2_		O_3_	
			
		%	*P* value^a^	%	*P* value^a^	%	*P* value^a^	%	*P* value^a^
(5–95)	5% L	0.49 (0.13, 0.86)	0.20	1.70 (0.82, 2.58)	0.18	1.48 (0.70, 2.26)	0.43	1.66 (0.28, 3.04)	0.17
	95% U	0.72 (0.16, 1.29)	0.11	1.45 (0.33, 2.57)	0.58	1.68 (0.61, 2.76)	0.36	0.88 (0.31, 1.46)	0.60
	5%–95%	0.26 (0.06, 0.47)		1.16 (0.58, 1.75)		1.22 (0.65, 1.79)		0.75 (0.25, 1.24)	
(10–90)	10% L	0.45 (0.13, 0.77)	0.20	1.43 (0.67, 2.19)	0.42	1.32 (0.61, 2.03)	0.73	1.81 (0.56, 3.05)	0.07
	90% U	0.80 (0.24, 1.36)	0.08	1.64 (0.49, 2.79)	0.36	1.83 (0.76, 2.90)	0.23	0.91 (0.33, 1.50)	0.51
	10%–90%	0.25 (0.05, 0.46)		1.15 (0.55, 1.76)		1.22 (0.65, 1.80)		0.74 (0.24, 1.23)	
(15–85)	15% L	0.51 (0.23, 0.79)	0.03^b^	1.63 (0.96, 2.31)	0.07	1.56 (0.92, 2.19)	0.15	2.57 (1.53, 3.62)	<0.01^b^
	85% U	0.30 (−0.17, 0.79)	0.75	0.64 (−0.46, 1.75)	0.42	0.91 (−0.0, 1.82)	0.46	0.62 (0.06, 1.18)	0.33
	15%–85%	0.23 (0.02, 0.44)		1.06 (0.43, 1.70)		1.22 (0.63, 1.80)		0.88 (0.37, 1.40)	
(20–80)	20% L	0.44 (0.20, 0.69)	0.08	1.66 (1.00, 2.31)	0.04^b^	1.59 (0.97, 2.20)	0.07	2.37 (1.45, 3.29)	<0.01^b^
	80% U	0.16 (−0.26, 0.60)	0.75	0.47 (−0.56, 1.50)	0.23	0.78 (−0.04, 1.61)	0.25	0.56 (0.04, 1.09)	0.10
	20%–80%	0.23 (0.01, 0.45)		1.06 (0.42, 1.69)		1.20 (0.62, 1.79)		0.98 (0.45, 1.52)	
(25–75)	25% L	0.38 (0.14, 0.63)	0.36	1.45 (0.80, 2.09)	0.57	1.44 (0.82, 2.05)	0.59	1.87 (1.05, 2.70)	0.02^b^
	75% U	0.13 (−0.27, 0.54)	0.47	0.49 (−0.50, 1.49)	0.09	0.87 (0.09, 1.65)	0.17	0.54 (0.04, 1.05)	0.07
	25%–75%	0.28 (0.05, 0.50)		1.28 (0.62, 1.94)		1.32 (0.73, 1.91)		1.03 (0.47, 1.59)	

Abbreviations: PM_10_, particulate matter less than 10 microns in diameter; SO_2_, sulfur dioxide; NO_2_, nitrogen dioxides; O_3_, ozone.

^a^*P* value for lower and upper temperature strata compared with normal temperature.

^b^Statistically significant (*P* < 0.05).

**Table 3.** Percent change (95% CI) in respiratory mortality per 10-µg/m^3^ increment in air pollutants at different temperature strata

Percentile cut-points for temperature (L, U)		PM_10_		SO_2_		NO_2_		O_3_	
			
		%	*P* value^a^	%	*P* value^a^	%	*P* value^a^	%	*P* value^a^
(5–95)	5% L	0.24 (−0.33, 0.82)	0.95	1.53 (0.11, 2.95)	0.74	1.30 (0.03, 2.57)	0.75	0.98 (−1.22, 3.19)	0.86
	95% U	0.22 (−0.74, 1.19)	0.94	1.64 (−0.28, 3.58)	0.92	1.05 (−0.80, 2.91)	0.64	0.63 (−0.38, 1.65)	0.72
	5%–95%	0.26 (−0.07, 0.60)		1.73 (0.74, 2.72)		1.46 (0.50, 2.42)		0.79 (−0.07, 1.67)	
(10–90)	10% L	0.37 (−0.13, 0.88)	0.56	1.57 (0.33, 2.80)	0.72	1.56 (0.40, 2.71)	0.70	2.34 (0.39, 4.29)	0.10
	90% U	0.23 (−0.74, 1.20)	0.99	1.65 (−0.36, 3.67)	0.90	1.10 (−0.76, 2.97)	0.74	0.42 (−0.62, 1.46)	0.33
	10%–90%	0.23 (−0.10, 0.57)		1.76 (0.74, 2.79)		1.39 (0.42, 2.36)		0.88 (0.00, 1.75)	
(15–85)	15% L	0.55 (0.11, 0.99)	0.04^b^	2.01 (0.90, 3.11)	0.26	1.82 (0.78, 2.87)	0.06	2.79 (1.13, 4.46)	<0.01^b^
	85% U	0.35 (−0.49, 1.19)	0.60	1.38 (−0.56, 3.33)	0.95	1.27 (−0.33, 2.88)	0.84	0.53 (−0.46, 1.53)	0.56
	15%–85%	0.13 (−0.22, 0.48)		1.44 (0.36, 2.53)		1.12 (0.12, 2.12)		0.81 (−0.09, 1.72)	
(20–80)	20% L	0.32 (−0.07, 0.72)	0.48	1.70 (0.62, 2.79)	0.99	1.55 (0.53, 2.57)	0.48	2.11 (0.60, 3.62)	0.04^b^
	80% U	0.38 (−0.38, 1.14)	0.62	1.62 (−0.19, 3.43)	0.92	1.28 (−0.18, 2.74)	0.97	0.64 (−0.28, 1.57)	0.82
	20%–80%	0.19 (−0.17, 0.56)		1.71 (0.61, 2.81)		1.30 (0.29, 2.31)		0.75 (−0.19, 1.69)	
(25–75)	25% L	0.51 (0.12, 0.90)	0.01^b^	2.16 (1.09, 3.23)	0.08	1.90 (0.88, 2.93)	0.02	2.31 (0.93, 3.69)	<0.01^b^
	75% U	0.14 (−0.56, 0.86)	0.79	0.73 (−1.01, 2.47)	0.52	0.72 (−0.63, 2.09)	0.51	0.46 (−0.43, 1.35)	0.45
	25%–75%	0.05 (−0.31, 0.43)		1.26 (0.12, 2.41)		1.10 (0.09, 2.12)		0.79 (−0.19, 1.77)	

Abbreviations: PM_10_, particulate matter less than 10 microns in diameter; SO_2_, sulfur dioxide; NO_2_, nitrogen dioxides; O_3_, ozone.

^a^*P* value for lower and upper temperature strata compared with normal temperature.

^b^Statistically significant (*P* < 0.05).

## DISCUSSION

In this community-based time-series analysis we found a statistically significant interaction between air pollution and lower temperature in their effects on daily mortality. Our findings were limited to PM_10_ and O_3_. Unlike previous studies,^26^^,^^27^ we did not find a significant interaction between air pollution and higher temperature.

Although the underlying mechanism is unclear, previous studies have shown that extreme temperature might increase the workload of the cardiopulmonary system and induce adverse cardiopulmonary events.^28^^,^^29^ For example, sudden inhalation of cold air was found to be associated with the release of inflammatory mediators associated with mast cells in a human study.^30^ In addition, marked changes in ambient temperature can cause physiologic stress and alter a person’s physiologic response to toxic agents, perhaps making them more susceptible to the effects of air pollutants.^31^^,^^32^ Moreover, most air pollution-related deaths occur in elderly adults,^33^ who have a lower capacity for thermoregulation^34^ and a higher sweating threshold as compared with younger persons.^35^ Therefore, an interaction between PM_10_/O_3_ and extreme temperature is biologically plausible.

As mentioned earlier, a number of studies,^10^^,^^12^^,^^13^ including our own,^11^^,^^14^ have investigated the interaction between air pollution and season. The present study did not investigate seasonality; instead, we focused on the related issue of the interaction between air pollution and extreme temperature, since temperature and season are associated. Our finding of a stronger association between air pollution and daily mortality on extremely cold days confirms those of 3 earlier seasonal analyses in Hong Kong, Shanghai, and Athens,^12^^,^^14^^,^^36^^,^^37^ but conflicts with those of several other reports that noted greater effects during the warm or hot season.^21^^,^^38^^–^^43^ In London, for example, the effects of NO_2_ and SO_2_ were stronger in the warm season than in the cool season.^38^ A combined analysis of data from 9 European cities also showed that SO_2_ had a slightly stronger effect during the warm season than during the cool season.^44^ For O_3_, which usually reaches higher concentrations in the warm season, several recent meta-analyses and multi-city analyses also found that the effect was evident only during the warm season.^39^^,^^41^^,^^43^

Our observation of stronger effects of air pollution on extremely cold days might be due to the larger sample size of daily numbers of deaths on cold days (Figure 1). Moreover, mean PM_10_ concentrations on cold days were higher than on days with normal or high temperatures. These 2 factors might have increased the power of the analyses of cold days. Because pollutant levels are correlated, the greater effects of air pollution observed on cold days may also be due to the effects of other pollutants that were also at higher levels on those days.^37^ Another potential explanation for the temperature-specific effects of PM_10_ is that levels of the most toxic particles might reach a maximum during the cool season in Shanghai. Unlike gaseous pollutants, the constituents of PM_10_ might vary by season as a complex mixture.^45^

Previous studies in both Wuhan^27^ and Tianjin^26^ showed that high temperatures might increase the health effects of air pollution. However, our analysis in Shanghai did not find a significant interaction between air pollution and extremely high temperature. We assume that exposure patterns contribute to various temperature-specific health effects in different cities. Shanghai receives considerable rainfall in summer. During those days of high temperatures, Shanghai residents tend to use air conditioning more frequently because of the higher temperature and humidity, thus reducing their indoor exposure. For example, in a survey of 1106 families in Shanghai, 32.7% never turned on an air conditioner during winter, as compared with 3.7% in the summer.^46^ Heavy rain in the summer may reduce time outdoors, thus decreasing personal exposure. In contrast, the winter in Shanghai is drier and less variable, so people are more likely to go outdoors and open windows.

The system for death coding may have had an impact on the effect estimates of air pollution on mortality.^47^ We did not consider deaths due to injuries or accidents because they are not believed to be associated with air pollution.^15^ Moreover, the causes of death for 2001 and 2002–2004 in Shanghai were coded according to the ICD-9 and ICD-10, respectively. However, a previous study in Wuhan showed that the change in ICD coding did not significantly affect the estimated effects of time-series studies of air pollution.^47^

Extreme temperature is related to global warming and other climate phenomena, such as El Niño. The possibility that extreme low temperatures intensify the health hazards of exposure to air pollution could spark new interest in the correlations between weather, air pollution, and health. Of course, our findings require replication, especially in areas with different weather patterns; but if substantiated, they provide insight into the health impact of both air pollution and climate change.
